# Microwave platform as a valuable tool for characterization of nanophotonic devices

**DOI:** 10.1038/srep35516

**Published:** 2016-10-19

**Authors:** Ivan Shishkin, Dmitry Baranov, Alexey Slobozhanyuk, Dmitry Filonov, Stanislav Lukashenko, Anton Samusev, Pavel Belov

**Affiliations:** 1 ITMO University, Metamaterials Laboratory, St. Petersburg, 197101, Russia; 2 Institute for Analytical Instrumentation of RAS, St. Petersburg, 198095, Russia

## Abstract

The rich potential of the microwave experiments for characterization and optimization of optical devices is discussed. While the control of the light fields together with their spatial mapping at the nanoscale is still laborious and not always clear, the microwave setup allows to measure both amplitude and phase of initially determined magnetic and electric field components without significant perturbation of the near-field. As an example, the electromagnetic properties of an add-drop filter, which became a well-known workhorse of the photonics, is experimentally studied with the aid of transmission spectroscopy measurements in optical and microwave ranges and through direct mapping of the near fields at microwave frequencies. We demonstrate that the microwave experiments provide a unique platform for the comprehensive studies of electromagnetic properties of micro- and nanophotonic devices, and allow to obtain data which are hardly acquirable by conventional optical methods.

Nanophotonics is a rich and rapidly developing field devoted to the engineering of light-matter interactions and control over the flow of the photons at the subwavelength scale^1^,^2^,^3^,^4^,^5^,^6^. The fundamental limitations of the semiconductor microelectronics, such as maximum operational frequency due to the energy dissipation can be potentially overcome with the use of all-optical circuits operated by nanophotonic devices^1^,^7^,^8^,^9^,^10^. However, the development of the nanophotonic devices requires enormous efforts. Despite the fact that the modern advanced fabrication techniques^11^,^12^ and novel materials^13^ allow to create a rich variety of nanostructures, including two- and three-dimensional metamaterials^14^,^15^,^16^,^17^ and photonic crystals^18^, the detailed characterization of such samples is still challenging^19^. Near-field scanning optical microscopy is one of the key tools for investigation of the properties of nanophotonic structures, enabling the mapping of electromagnetic fields at the nanoscale^20^,^21^. However, the nanoscale mapping of the field is perturbated by the presence of relatively large collecting probe. Furthermore, the direct interpretation of the signal collected by the probe is a formidable task, especially in the case of strong near-field interaction between the sample and the probe^22^,^23^. Finally, the information about the phase of the electromagnetic signal, which can be crucially important for certain applications, in most cases remains unavailable and requires the most complex setups^21^,^24^. While the near-field scanning optical microscopy remains exceptional cutting-edge technique for characterization of structures at the nanoscale^19^, other ideas, inspired by the scalability of Maxwell equations, can be involved for optimization and partial characterization of nanophotonic devices.

The macroscopic objects are better candidates for study and optimization due to the ease of fabrication and measurement techniques, which opens access to all-electromagnetic characteristics of the structure response. The microwave range offers one the opportunity for studies of up-scaled counterparts of nanophotonic structures. The larger wavelength allows to use mm and cm sized samples and well-developed near field scanning technique. Importantly, in this frequency range the size of the probe is much smaller than the operational frequency, which guarantees minimal perturbation of the near-field, while the usage of different kinds of probes (magnetic or electric) allows to map both amplitude and phase of different components of magnetic and electric fields correspondingly. The Maxwell’s equations scalability has already been used in several notable cases, such as demonstration of the concept of photonic crystals^25^, the study of properties of nanoantennas^26^, subwavelength waveguides^27^, oligomers^28^, hyperbolic metasurfaces^29^, topologically protected edge states^30^, and studies of nonlinear metamaterials^31^.

It is worth mentioning, that in general case one may expect the same electromagnetic response of micro- and macro- structures only if the proper selection of materials is done. In case of non-dispersive materials the same material properties (permittivity and permeability) in the respective spectral ranges should be chosen. One has to note, that in general the material parameters of a given substance can differ significantly in microwave and optical domain, which does not allow direct scaling of the structure without a lookup for the analogues. Thus, finding a material with pairing parameters for the prototyping can be challenging since a suitable pair should be looked for in every individual case.

In this Report we propose a microwave platform as a valuable tool for characterization of nanophotonic devices. We have chosen an add-drop filter (ADF) based on a ring resonator as the simplest example of the well-known photonic device. The ADF has been studied in details in optics and found applications in variety of areas, such as optical sensing, telecommunications, optical interconnects and signal processing^32^,^33^,^34^,^35^. The microring-based ADFs exhibit narrow resonances and are prospective devices for higher-order filters and wavelength division multiplexion applications^36^,^37^. Here, in order to outline the advantages of the microwave platform, we have developed two ADF prototypes for optical and microwave frequency ranges. We have used direct laser writing and computer-controlled milling for the fabrication of microscale and macroscale samples, respectively. We have performed optical and microwave measurements of both prototypes and directly show the major advantages of the microwave platform. In particular, we show the non-perturbated near-field maps of the amplitude and phase of the electric field of the ADF operated in various regimes. The proper selection of the ring and waveguide dimensions allows us to limit the amount of excited modes in the cavity. Thus, we believe that our approach will allow to minimize the efforts during the optimization of nanophotonic structures and simultaneously give access to broad range of electromagnetic characteristics.

## Results and Discussion

**Figure 1:**
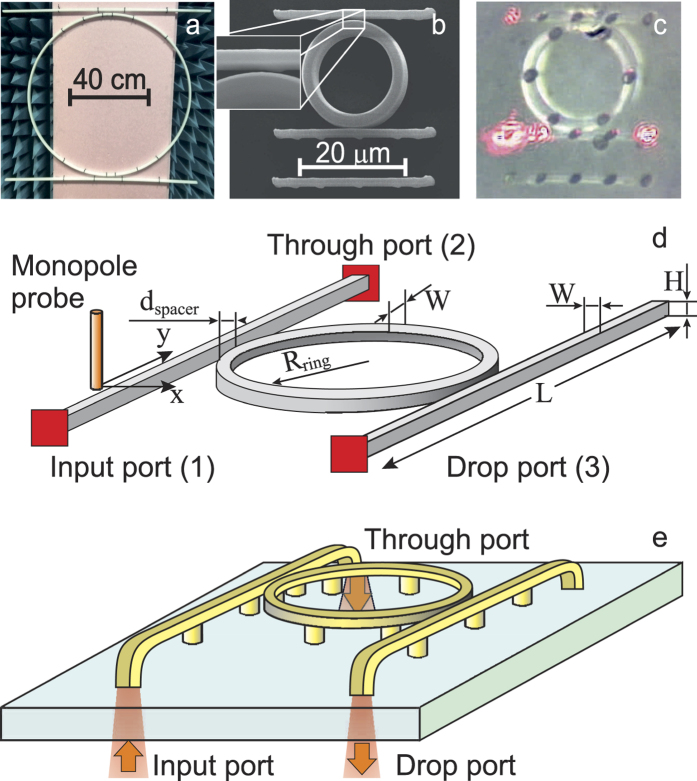
Microwave and optical add-drop filters. (**a**) The microwave prototype overview. Waveguide width W = 9.5 mm, waveguide height H = 20 mm, ring radius R_*ring*_ = 400 mm, separation between waveguide and ring d_*spacer*_ = 9.5 mm; waveguide length L = 1000 mm. The waveguide port is used as an excitation source. **(b)** The SEM image of optical ADF with 300 nm gap between the ring and the waveguides. **(c)** The microphotograph of optical ADF taken during the measurements. Waveguide width W = 1.5 *μ*m, waveguide height *H* = 1 *μ*m, ring radius R_*ring*_ = 10 *μ*m, separation between waveguide and ring d_*spacer*_ ≈ 300 nm; waveguide length L = 30 *μ*m. The measurement schemes of microwave and optical filter prototypes are shown in (**d,e**) respectively. The through and drop channels both for microwave and optical measurements are marked in the schemes.

We have considered two systems – an optical ADF and a microwave one. The images of fabricated samples and measurement schemes are depicted in Fig. 1. The materials, which were used for fabrication of both ADFs, had similar values of dielectric permittivity in respective frequency ranges. The microwave ADF was made from polyethylene, which is characterized by permittivity *ε* = 2.23 with the loss tangent, less than 10^−3^ in microwave region^38^, and placed on styrofoam substrate(see Fig. 1(a)). It is important to note, that in microwave spectral range it is convenient to use styrofoam material as a substrate/holder due to the fact that it is almost transparent and characterized by *ε* = 1.05. This allows to position the sample without unwanted influence of the substrate and provides access to easy characterization of the prototype, which resides effectively in free space. The macro-ADF was formed from a ring resonator with diameter of 0.8 m coupled with two one-meter long waveguides placed on both sides of the ring, which served as through- and drop-channels (see Fig. 1(a)). Direct laser writing setup has been used for fabrication of a dimensionally scaled down ADF^39^. The permittivity of the photoresist used for fabrication of optical ADF coincide with the permittivity of polyethylene in microwave.

**Figure 2:**
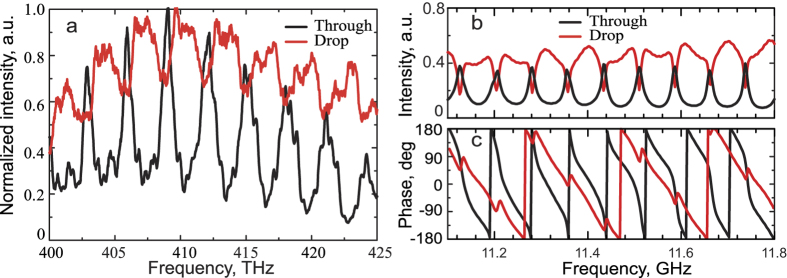
Experimental spectra. (**a**) The measured signal in through and drop channels of the optical microring ADF (normalized to the maximum intensity). **(b)** Measured S-parameters of a microwave ADF (normalized to the reference waveguide S-parameters), S21 corresponds to “Through” data, S31 corresponds to “Drop” data; **(c)** respective phases of the measured signals.

We have carried spectral characterization of the micro-optical ADF: the transmission in “through” and “drop” channels was measured using the scheme depicted in Fig. 1(e). Rather than using tapered fibers for excitation of microring, the light was focused to the end of the bent waveguide and collected using a spectrometer with the motorized pinhole, which allowed to spatially select the region of spectral data acquisition (for further details see Methods). The measured through and drop channel spectra of the micro-optical ADF are shown in Fig. 2(a). We can observe clearly distinguishable dips in “through” and peaks in “drop” channel signals.

The relatively large thickness (W) of the ring and the waveguides compared to the wavelength of the light in the measured region, results in simultaneous excitation of several higher-order modes in the waveguide and the microring. The observed spectra in both channels could be considered as an envelope of different modes supported by the waveguide (for more details see the supplementary material). The contributions of these individual modes result into broadening of peaks and dips in transmission spectra (see Fig. 2(b)). Therefore, the spectra clearly demonstrate the filtering capabilities of the fabricated ADFs. The simultaneous presence of several modes at a given frequency makes the direct analysis of the mode structure impossible without supporting numerical simulations basing only on the field distribution maps.

On the other hand, by choosing the dimensions of the resonator for the microwave ADF it is possible to make it support only a single mode at the frequency range under study. Such model system allows to investigate the properties of the excited whispering gallery mode without the necessity to distinguish the modes with different azimuthal and radial indexes. The measured S-parameters S21 and S31, are equivalents of transmission measurements of input - through (through) and input - drop (drop) channels of an optical ADF correspondingly. The waveguide ports were attached to the dielectric waveguides of the ADF as depicted on the Fig. 1(d). The measured values of S21 and S31 are presented in Fig. 2(b). The general trend of the graphs can be easily seen – the numerous peaks in S31 which correspond to dips in S21 appear. Both micro-optical and microwave ADF demonstrate the excitation of the whispering gallery modes in the resonators.

The full width at half maximum of 11.434 GHz resonance was found to be 9.66 MHz. It corresponds to the system’s loaded Q-factor of ≈1200, which was estimated by Lorentzian fitting of the resonance peak. The data on the phase of the measured electric field is given on Fig. 2(c). The fact, that the phase shift occurs only once on the resonance frequency confirms, that only the mode with lowest axial and radial numbers is excited.

Next we proceed to the analysis of the near-field measurements. Near-field profiles of microscale whispering gallery mode cavities have been already studied to a certain degree by several groups. However, comprehensive analysis of near-field profiles of micro- and nano-ADFs and individual resonators can be complicated. Moreover, undesired effects mediated by substrate and sample-tip interactions, may lead to unclear experimental results. On the other hand, the microwave platform allows to fully mimic micro- and nano-scale experiments and map real and imaginary parts of particular electromagnetic field components. To demonstrate this, we have performed near-field mapping of macro-ADF. Experimental setup is shown on the Fig. 1(d). The electric field probe has been mounted on three dimensional automatic near-field scanner, that gives opportunity to measure electric field in the vicinity of the structure as a function of coordinates.

**Figure 3:**
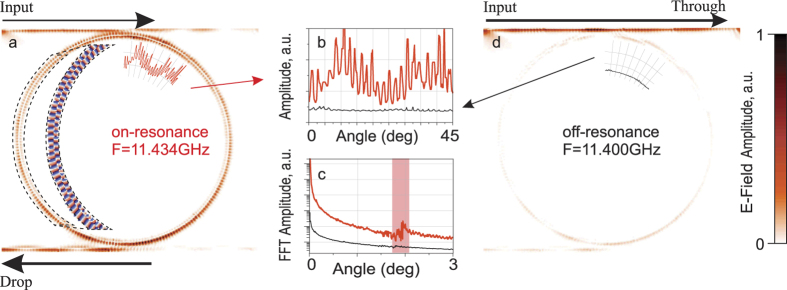
Measured near-field distributions of E_*z*_ component of electric field in an add-drop-filter. **(a)** – on resonant frequency of the ring and **(d)** – off resonance. The inset in **(a)** shows the measured phase distribution for the area marked with dotted line. **(b)** Intensity distributions were extracted along the curved path for both on- and off-resonance field distributions. **(c)** The fast Fourier transforms of the measured intensity distributions.

Figure 3(a,d) shows the measured electric field spatial distributions on resonant and non-resonant frequencies respectively. High resolution capability of microwave imaging is clearly observed. The intensity of electric field is oscillating in accordance with the standing wave field distribution in a polymer ring at resonance frequency [Fig. 3(a) *f* = 11.434 GHz], while off-resonant field distribution demonstrates no oscillating behavior. Figure 3(b) demonstrates the angular cross sections of the E-field distributions, and in Fig. 3(c) we plot the fast Fourier transform of the cross sections. This allows us to estimate the angular mode number (N = 92) from the periodic beatings with angular spacing between adjacent maxima of 1.955°.

The on-resonance field distribution is shown in Fig. 3(a) for the frequency of 11.434 GHz. The electric field probe not only allows us to capture the amplitude distribution of the field components, but also gives us the information on the phase of the fields. So far, we can observe, that a standing wave is formed in the ring, which can be clearly seen both in amplitude and phase images.

This feature offers the significant advantage of the microwave measurements over NSOM due to the possibility of direct field measurements. Moreover, the electric field probe is dominantly receiving the information on the certain component of the electric field, while the magnetic probe - on the magnetic one. This is important point, since in the NSOM measurements additional data processing techniques have to be employed in order to properly interpret the measured signal, while the microwave platform allows to obtain direcly the information on the amplitude and phase of the electric/magnetic field. Finally, with the proper orientation of the probe, different components of the measured electric/magnetic field can be retrieved.

## Conclusion

In this work, the microwave platform was demonstrated as a valuable tool for characterization of nanophotonic devices. The ring resonator based add-drop filter, whose properties are already well-studied, was used as the model object. The two prototypes of the ADF in optical and microwave domains were fabricated using milling and direct laser writing and their transmission properties were measured. It was shown, that the microwave platform can be used as a model object to study complex electromagnetic phenomena like whispering gallery modes. The microwave platform can offer certain advantages in the studies of micro- and nanophotonic systems in terms of ease of the measurements of the electromagnetic fields.

## Methods

### Microwave prototype fabrication and characterization

The microwave ADF prototype was milled from the sheet of Orbilan PE-500 high-density polyethylene with precision of 0.1 mm. The measurement scheme is shown in Fig. 1(b). The waveguide dimensions (20 mm × 9.5 mm) were selected basing on the excitation port dimensions. The ring diameter (80 cm) was chosen to minimise the losses due to the scattering on the cavity sidewalls. The ring and the waveguides were mounted on a styrofoam sheet, whose permittivity is close to unity within frequency range under study. For the S-parameter measurement a pair of WR-90 waveguide ports (22.86 × 10.16 mm^2^ cross-section, operating in X-band, 8.5–12 GHz) was used. The S-parameters have been measured using an E8362C Vector Network Analyzer. The mapping of the electric field distribution was performed using a monopole probe (2 mm diameter) and two-dimensional mechanical near-field scanner, the system was excited by the waveguide port connected to the VNA. The scans were taken 5 mm above the surface of the filter.

The dimensions of the waveguide were sub-optimal for proper guiding in the studied frequency range, which resulted in significant insertion losses at frequencies lower than 10 GHz.

### Optical prototype fabrication and characterization

The fabrication of an optical ADF was carried by direct laser writing process (DLW). This fabrication method is in principle quite similar to a macroscopic 3D-printing and uses the photocurable resin to form the microscale objects from it. A hybrid organic-inorganic material based on zirconium propoxide was used as a photoresist with Irgacure 369 (Ciba Specialty Chemicals Inc., USA) as a photoinitiator. The material exhibits low shrinkage upon development^40^. A commercial DLW setup was used for the fabrication of microstructures (Laser Zentrum Hannover, Germany). A Ti:Sapphire laser (TiF-100F, Avesta Project, Russia) was used as a source of femtosecond pulses with duration of 50 fs centered around 790 nm seeded with the repetition rate of 80 MHz. The laser radiation was focused into the volume of photoresist using high-NA oil immersion objective (NA = 1.4, Carl Zeiss MicroImaging GmbH, Germany). The photoresist was drop-casted on 170 *μ*m thick glass coverslide used as a substrate and was dried at 70° for one hour.

The microring and the waveguides composing the ADF were formed by continuous scanning of the focused laser beam through the volume of the photoresist. The average laser power was set to 8 mW, the scan speed was set to 30 *μ*m/s. The ring diameter was chosen to be (d = 20 *μ*m). The width of the ring obtained from SEM was W = 1.5 *μ*m. The SEM-image of the fabricated ADF is shown in Fig. 2(a). The width of the ring and the waveguide width were chosen in order to provide stability to the ADF during development stage. In order to reduce mode leaking to the substrate and to prevent sticking of the ring and the waveguides to the glass, they were supported by micropillars. Therefore, we have found, that micropillars reduce the coupling efficiency between the waveguide and the microring and might also result in reduction of Q-factor of the cavity.

The characterization of the optical properties of the ADF was carried out as shown in Fig. 1(c,e). The excitation of the ADF was performed by a supercontinuum light source (Fianium WhiteLase SC400-6) combined with tunable band-pass filter (Fianium SuperChrome) focused by a microscope objective lens to the back aperture of the input waveguide. The coupling of the light to the microring was achieved by fabricating a free-standing waveguide side-coupled to the resonator. A reflection microscopy setup [see Fig. 1(e)] was used to acquire the spectra: the excitation and collection spots were spatially separated, which allowed to perform transmission measurements of through and drop channels of the filter separately. The collected light was analyzed with a spectrometer (Horiba LabRam HR) in confocal arrangement.

## Additional Information

**How to cite this article**: Shishkin, I. *et al*. Microwave platform as a valuable tool for characterization of nanophotonic devices. *Sci. Rep.* **6**, 35516; doi: 10.1038/srep35516 (2016).

## Supplementary Material

Supplementary Information
