# Supplementary material for: Microwave platform as a valuable tool for characterization of nanophotonic devices
Source: Sci Rep. 2016 Oct 19;6:35516. doi: 10.1038/srep35516 (PMC5069494; doi:10.1038/srep35516)
Supplement: Supplementary Information [file srep35516-s1.pdf]

Electronic supplementary information

Microwave platform as a valuable tool for characterization of nanophotonic devices

Ivan Shishkin[1*], Dmitry Baranov[1], Alexey Slobozhanyuk[1], Dmitry Filonov[1],

Stanislav Lukashenko[1, 2], Anton Samusev[1], and Pavel Belov[1]

[1]ITMO University, Metamaterials Laboratory, St. Petersburg, 197101, Russia

[2]Institute for Analytical Instrumentation of RAS, St. Petersburg, 198095, Russia

[*]Correspondence should be addressed to Ivan Shishkin
E-mail address: i.shishkin@metalab.ifmo.ru

Supplementary information

1. Modes supported by the optical add-drop filter

Large thickness of the fabricated optical ring resonator and the waveguides compared to the wavelength of the light in the measured spectral domain result into simultaneous excitation of several higher-order modes in the waveguide and the microring. The measured spectra can be considered as an envelope of different modes supported by waveguide. The contributions of individual modes result into broadening of the peaks and dips in transmission spectra of the ADF. The spectra of individual modes supported by the waveguide and their field distributions are presented in Figure S1.

The peaks in the spectra corresponding to the modes with neighboring indexes exhibit a small spectral shift of resonance frequencies with respect to each other. The resulting spectrum can be considered as an envelope function of the modes excited simultaneously in the waveguide with respective weight coefficients. Those coefficients are defined by efficiency of coupling of light from free space to the mode propagating in the waveguide, which can be calculated as an overlap integral between the free-space beam field distribution and mode field. Since we aligned our sample to achieve the maximum possible signal on the other end of waveguide, we can assume, that the most efficient coupling is achieved between the excitation beam (which is considered Gaussian) and the lowest order mode, which can exist in waveguide. All higher-order modes have lower coupling efficiency. This results in their smaller contribution to overall spectral response of the ADF. The summed up spectra with the proper weight coefficients are presented on Figure S1(c).

2. Influence of the posts on the optical properties of the ADF

The introduction of posts to the optical ADF was dictated by the need of stability of the microstructure

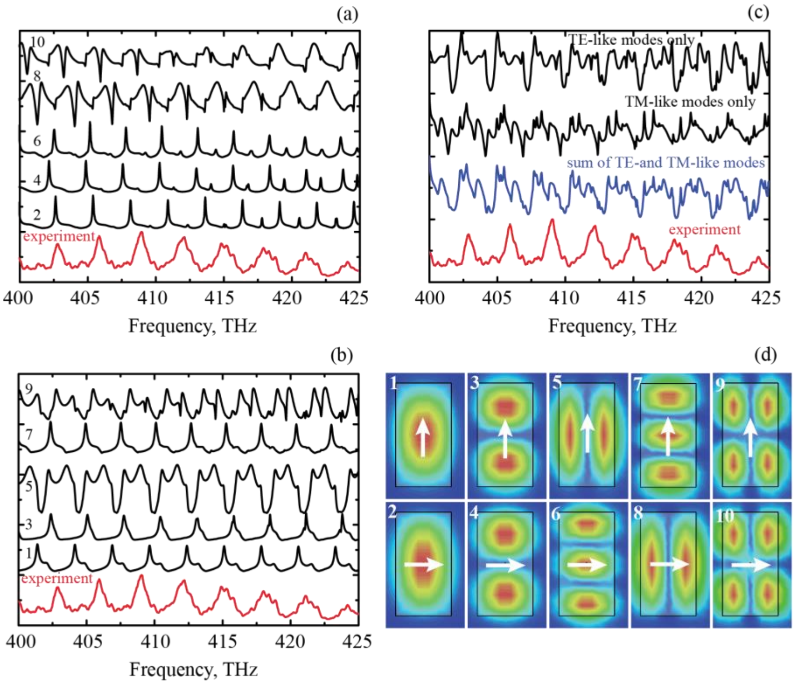

Figure S1. Comparison between measured and simulated optical "Drop" channel spectra. (a) TE-like modes spectra (b) TM-like modes spectra (c) comparison between calculated total spectra composed of only TE-like modes, only TM-like modes and their sum with the measured spectrum (d) electric field distribution and polarization of the modes supported by the waveguide. The field maps are calculated at the frequency of 412.5 THz.

during development of photoresist. These posts also influence the system behavior. First, they provide polarization sensitivity for lowest-order TM-like modes, which are suppressed due to presence of the pillars, see Fig. S2(a,b). Second, the pillars act as additional scatterers for the modes propagating in the waveguide, resulting into the broadening of the peaks in the spectra. The simulated field distributions at the resonance and off the resonance frequencies are shown in Figure S3.

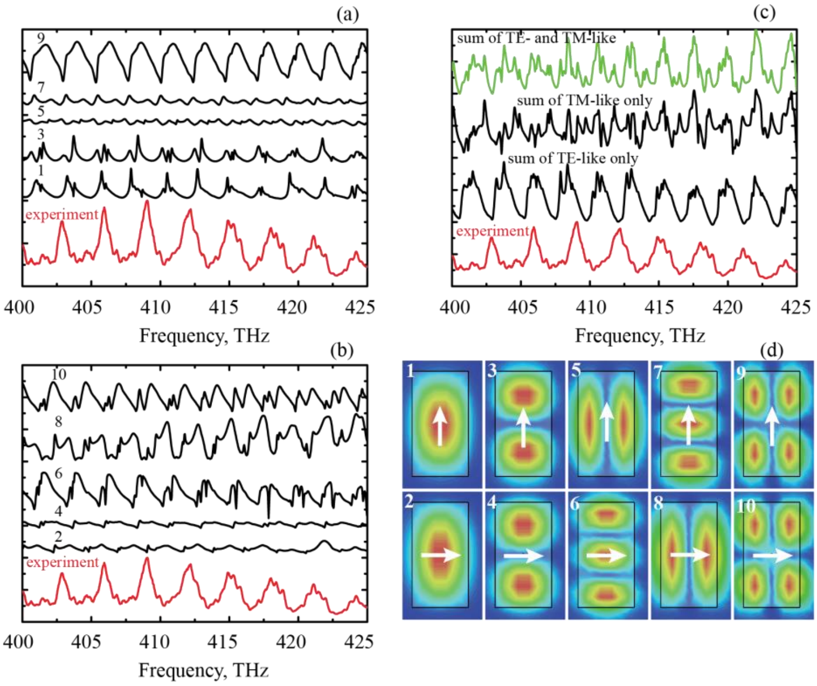

Figure S2. Comparison between measured optical "Drop" channel spectra and simulations of ADF system with support pillars. (a) TE-like modes spectra (b) TM-like modes spectra (c) comparison between TE-like and TM-like and their sum with the measured spectra (d) electric field distribution and polarization of considered waveguide modes. The field distributions were calculated at the frequency of 412.5THz

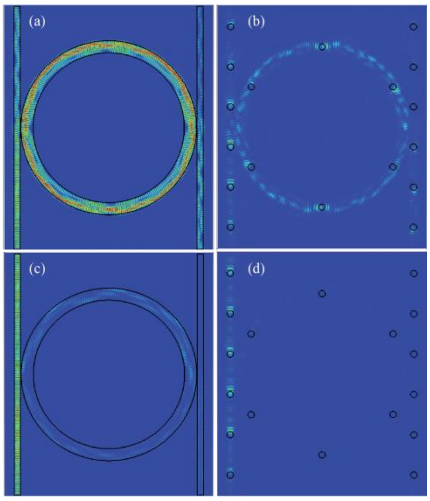

Figure S3. (a,b) calculated electric field distribution in ADF at the resonance (at the frequency of 412.7 THz). The field cross-sections are taken across the ring(a) and 200 nm below the ring(b). (c,d) calculated electric field distributions off-resonance at 414.27 THz. Cross-sections taken across the ring(c) and 200nm below the ring(d).
